# Loot

**Published:** 1869-04-15

**Authors:** 


### LOOT.

*L' Univers Médicale* contains the following results of experiments upon the solubility of dipotheritic membranes. Subjected for an hour to the vapor of sulphate of mercury false membrane was not dissolved, and when subjected to the action of a concentrated solution of pepsine, its solution was effected only after twelve hours, which period was shortened to *eight minutes* by the addition of six to ten drops of lactic acid. Neither sulphuric, hydrochloric or nitric acids accomplish the desired result. Acetic and citric acid render diphtheritic membrane transparent but do not dissolve it completely. Two drops of lactic acid in five grammes of water dissolved twenty centigrammes of tough membrane in three minutes. Lime water produced the same result still more rapidly; lactate of lime is inert- Weak solutions of soda and potassa dissolved the membrane in eight or ten minutes, and better than when con. centrated. Nascent bromine and bromine water only disintegrate the membranes; bromide of potassium and the sulphates, bicarbonates and nitrates of soda and potassa. Chloride of zinc and chromic acid are without action. The chlorates of potassa and soda dissolve membranes but slowly.

---

### *Monstrosity.*

Dr. O. C. Gibbs, of Frewsburg, N. Y., reports to the *Lancet and Observer* the birth of a monstrosity characterized by the absence of cranial and spinal bones, of the entire integument, and of the spinal muscles from the third cervical to the fifth lumbar vertebra, also of the anterior muscles; the liver, stomach and heart being exposed. The brain, enclosed in its membranes, hung suspended down the back.

---

### *Fragilitas Ossium.*

Dr. Joseph Jones, of the University of Louisiana, reports to the St. Louis *Med. Archives* the case of a dark mulatto who had sustained, from the most trivial accidents, fractures of the extremities to the number of fifty, by reason of which he had become completely dwarfed and misshapen, and unable to walk except with the use of crutches. The parents were healthy, and the condition of their son was attributed by them to the results of an attack of typhoid fever which was treated with large doses of calomel, which the Doctor does not endorse. Dr. J. indicates the following points of interest in this case.

1st.—The painless character of the fractures.

2d.—The integrity of the general health, and the absence of allevidences of syphilitic or scrofulous cachexia or taint.

3d.—The constitutional origin and hereditary character of the disease was indicated by the brittle state of the bones in a cousin of the subject.

### *Prolapsed Funis.*

Dr. Yarnall reports to the St. Louis *Med. Archives* fifteen cases of prolapsed funis occurring in the practice of Dr. Papin, of that city, in which reduction was accomplished by placing the patient upon her elbows and knees, the pelvis being elevated, and passing the loop within the womb, beyond the promontory of the sacrum, and retaining it there until the head was again engaged in the superior strait. Of the fifteen cases ten were born alive, and five died, one from compression of the cord by the forceps, one from severing of the cord by the same instruments, a third from the two free administration of ergot, and the remaining two from impossibility of retaining the replaced cord, in consequence of the transverse position of the fætus.

---

### *Monstrosity.*

Dr. Steele recently presented to the St. Louis Medical Society a double headed monstrosity; a large and well-developed double-headed male child, having two entire and separate vertebral columns; two sacra separated by a rudimental mass of bone; a single pelvis; two legs; three arms, the third or middle one between the heads having one humerus, (double-headed, *i. e.* having two artieular facets,) one ulna, two radii, and two hands, (the palms approximating,) with two thumbs and seven fingers, the little fingers being merged into one, one bifurcated sternum, the ribs of the opposite sides articulating with it; the posterior ribs fused, making a median line resembling a third vertebral column; four scapulæ, the two median being confluent at their superior angles; two normal stomachs, with intestinal canals distinct for their upper two-thirds, and merged and single for the lower third; one rectum, one anus; two pairs of lungs and two hearts normal, one liver formed of two right lobes fused and contained in a common investment; two gall bladders, the right rudimentary; two spleens, one to the right, anterior, and the other to the left, posterior, in the thoracic cavity, into which they had escaped from the abdomen through an opening, one inch and a half in diameter, in the left leaflet of the diaphragm; two kidneys; one bladder and one penis.—*St. Louis Med. Arch.*

